# SNR Prediction with ANN for UAV Applications in IoT Networks Based on Measurements

**DOI:** 10.3390/s22145233

**Published:** 2022-07-13

**Authors:** Caio M. M. Cardoso, Fabrício J. B. Barros, Joel A. R. Carvalho, Artur A. Machado, Hugo A. O. Cruz, Miércio C. de Alcântara Neto, Jasmine P. L. Araújo

**Affiliations:** Electrical Engineering Graduate Department, Federal University of Pará, Belém 66075-110, Brazil; fbarros@ufpa.br (F.J.B.B.); joel.carvalho@itec.ufpa.br (J.A.R.C.); artur.machado@itec.ufpa.br (A.A.M.); hugo.oliveira@itec.ufpa.br (H.A.O.C.); miercio@ufpa.br (M.C.d.A.N.); jasmine@ufpa.br (J.P.L.A.)

**Keywords:** artificial neural network, communication channel, densely wooded, LoRa, signal-to-noise ratio, measurements

## Abstract

The 5G deployment brings forth the usage of Unmanned Aerial Vehicles (UAV) to assist wireless networks by providing improved signal coverage, acting as relays or base-stations. Another technology that could help achieve 5G massive machine-type communications (mMtc) goals is the Long Range Wide-Area Network (LoRaWAN) communication protocol. This paper studied these complementary technologies, LoRa and UAV, through measurement campaigns in suburban, densely forested environments. Downlink and uplink communication at different heights and spreading factors (SF) demonstrate distinct behavior through our analysis. Moreover, a neural network was trained to predict the measured signal-to-noise ratio (SNR) behavior and results compared with SNR regression models. For the downlink scenario, the neural network results show a root mean square error (RMSE) variation between 1.2322–1.6623 dB, with an error standard deviation (SD) less than 1.6730 dB. For the uplink, the RMSE variation was between 0.8714–1.3891 dB, with an error SD less than 1.1706 dB.

## 1. Introduction

The Number of Internet of Things (IoT) applications tends to further increase with the 5G extension across the globe. The technology will offer services such as massive machine-type communications (mMTC), which supports 100 times more devices per unit area than 4G, sending small data payloads periodically [1]. However, there are regions where 5G signals will be intermittent, low quality, or even unreachable, increasing the deployment and maintenance costs for IoT applications.

The Long Range Wide-Area Network (LoRaWAN) is an IoT protocol that uses Chirp Spread Spectrum (CSS) modulation. The spread spectrum technology is well-known for its low signal power level and higher spectral redundancy. Moreover, the signal level in spread spectrum applications operates below the noise floor, which means, to transmit information free of error considering a fixed Signal-to-Noise Ratio (SNR) value, only the signal bandwidth needs to be increased [2].

In LoRaWAN transmission, the CSS uses the Spreading Factor (SF) parameter, which plays a vital role. Increasing the SF value is the same as Increasing the signal bandwidth. Additionally, higher SF values mean an extension in range and higher receiver sensitivity (SNR values up to −20 dB). However, the payload size is reduced for higher SF, and transmission time on-air takes longer [3].

The LoRaWAN protocol, characterized by low power consumption, low data rate, and low cost, fulfills different IoT application requirements and already works in many services, such as farming inspection, security monitoring, and healthcare [4]. Moreover, 5G cellular IoT systems have limited maximum scalability and cannot fully satisfy the 5G mMTC target, and due to the aforementioned factors, the LoRaWAN protocol is a feasible solution that can contribute to future 5G-IoT non-time-critical sensor applications [5].

Unmanned Aerial Vehicles (UAVs, also known as drones) are another technology applied in the aforementioned services [6], and along with 5G, they are expected to increase the resilience of communication networks in emergency cases, that is, natural disasters. In [7], the authors developed an autonomous system to deploy UAVs for communication recovery during disasters and wide-area relief. Hence, UAV will help to improve network coverage in these cases and in situations where signals deteriorate because of many obstacles [8].

In this way, Long-Range (LoRa) and UAV are complementary technologies that can work together to improve 5G mMTC services and network coverage. Thus, it is necessary to understand how UAVs affect LoRa signals and their behavior through a communication channel. There are two traditional methods to analyze the communication channel: deterministic methods, such as Ray-Tracing (RT), which require a thorough model of the environment, and Stochastic methods based on exhaustive propagation measurement campaigns with different methodologies [9].

Therefore, this study analyzes the SNR for the LoRa technology communication channel at 915 MHz between a UAV and a moving receiver in a suburban densely wooded environment using ANN. Furthermore, the SNR use also considers the LoRa adaptive data rate technology (ADR), which adjusts SF accordingly with channel conditions [10]. Finally, a prediction model based on these measurements is proposed and compared with regression models generated with the same measurements.

**Figure 1:**
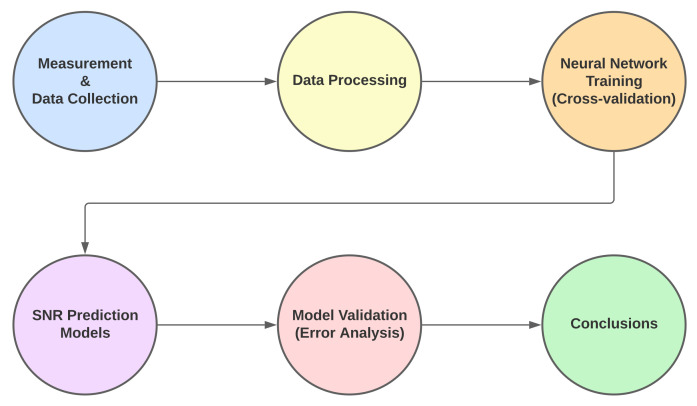
Workflow.

Figure 1 shows the necessary steps for the work completion, starting from measurement and data collection, which will describe the scenario and equipment used. Next is the Data processing module, in which only the relevant data to be used in the models is selected. Following that, is the ANN training in which the processed data are used as input and output sets. After, the development of baseline models, based on regression models using the data processing results. Finally, the model comparison is followed by the work’s conclusions.

The contribution of this study can be summarized as follow:

1. Verify the necessity of downlink and uplink analysis due to the reciprocity principle [11].
2. SNR downlink and uplink measurement campaigns using a UAV to understand how the signal behaves in a suburban and densely forested environment, considering both Line-of-Sight (LOS) and Non-Line-of-Sight (NLOS) situations.
3. Different SNR regression models and an ANN-based prediction model were established to represent the signal within Amazon cities, which could be reproduced in similar environments. Additionally, the proposed models could assist in improving LoRa ADR technology.

The remainder of this paper is organized as follows: Section 2 shows current state-of-the-art, Section 3 presents our study methodology, measurement campaigns, and collected data, followed by Section 4, which presents the results, and Section 5 presents the conclusions.

## 2. Related Works

In [12], the authors studied the use of UAVs in wireless networks, investigating the key challenges, applications, and fundamental open problems. They also presented UAV-enabled wireless networks challenges and the need for more realistic UAV channel models covering diverse environments and weather conditions. Furthermore, they indicate the necessity for Aerial-to-Ground (A2G) small-scale fading and UAV-UAV communications models.

In [13], the authors presented a comprehensive survey on measurement-and simulation-based radio propagation channel (RPC) modelling for low-altitude and UAV-enabled wireless networks, considering both downlink and uplink scenarios. The authors also described open research gaps, use cases, and the main problems. For example, UAV RPC modelling is limited to open areas with LOS links, at very low altitudes, and provides limited large-and small-scale fading analysis. Furthermore, the authors demonstrated the importance of studying UAV mobility and hovering, transmitting and receiving in both LOS and NLOS links.

In [14], the authors designed a flying gateway that aimed to extend the IoT gateway’s terrestrial coverage. First, they used a LoRa gateway and interface Long-Term Evolution (LTE) integrated into UAVs. Next, an endnode LoRa sent data as the received signal strength indicator (RSSI), humidity, and temperature in the uplink channel. Finally, the LoRa gateway sent data received via LTE interface to be displayed and analyzed in a server. The findings of this study demonstrate the feasibility of combining LoRa, UAV, and LTE to increase signal coverage in areas where terrestrial gateways do not reach. However, the authors did not analyze the communication channel or downlink scenario.

The author’s goal in [15] was to implement a system to monitor crops and farms using a flying LoRaWan gateway. They conducted two different tests: one in a parking building, and the other in a tree farm. The first showed a decrease in communication speed as the drone moved away from nodes horizontally. The second, i.e., in a tree farm, multiple connections between the Gateway and many endnodes were feasible. However, as in the study [14], authors in [15] analyzed only the uplink scenario.

In [16], the authors proposed a radio propagation model for a LoRaWAN network. They studied the Beirut-LB city environments by measuring RSSI and SNR for indoor, outdoor, urban and rural zones at 868 MHz using the SF 12. The results demonstrate the reliability of the LoRaWAN network for numerous IoT applications, reaching 9 km of coverage for dense urban areas and up to 47 km for the rural case, with a 90% packet delivery ratio threshold for both. However, the authors did not modify the SF, considering only the lowest data rate (DR), which in some cases might not be viable. In addition, uplink communication was the only one analyzed.

The authors in [17] studied the LoRa communication behaviors for indoor, outdoor-to-indoor, and urban environments, also analyzed the antenna polarization effects on transmission. For the urban environment, drones were utilized to increase transmission coverage. The authors discovered that drone height and antenna orientation are crucial in receiving signal strength (RSS). Drone heights of 25 and 50 m do not significantly impact RSS, but if the transmitting antenna is vertical, the received signal becomes stronger. However, the author used only one SF and restricted the work to examining uplink communication.

Most of the aforementioned studies considered only one propagation direction, either downlink (gateway to endnode) or uplink (endnode to gateway). In certain situations, unidirectional propagation modelling may represent communication channels in both ways owing to the reciprocity principle [11]. However, in this study, the propagation path between the transmitter and receiver contained a blend of open areas, buildings, and densely forested areas, that is, the terrain was not isotropic [18], and the reciprocity principle could not be used. Hence, it is relevant to conduct a comprehensive RPC study considering LOS and NLOS for UAV-enabled wireless networks in the most diverse cases.

Another approach is to use heuristic techniques for channel modelling using artificial neural networks (ANN), which are powerful tools capable of approximating any function [19].

In [20], the authors measured the received power at 521 MHz for a hybrid city-river environment to predict the electric field through an ANN. The ANN was also used in [21], in which the authors measured RSS to propose a path loss model for three different frequencies: 900 MHz, 1800 MHz, and 2100 MHz.

In addition, the authors in [22] proposed a propagation model for heterogeneous networks. They trained an ANN to predict the path loss using the measured received power at the following frequencies: 450, 850, 1800, 2100, and 2600 MHz. Moreover, ref. [23] used LoRa technology to measure RSSI for a downlink scenario at 868 MHz in a tropical environment and utilized an ANN to model path loss.

The works mentioned above proposed ANN models to characterize the environment and compared their results with classical propagation models, such as Okumura-Hata, Lee, and SUI. Their results showed the better performance of the ANN, proving its efficiency in predicting path loss in diverse circumstances.

Therefore, this work motivation is the need for more realistic UAV RPC models, considering both LOS and NLOS scenarios and the lack of uplink and downlink analysis in the same environment for the LoRa technology with different SF values.

## 3. Methodology

This section describes the proposed methodology, measurement campaigns that were conducted, uplink and downlink data analysis, and neural network configurations.

### 3.1. Measurement Campaign

**Figure 2:**
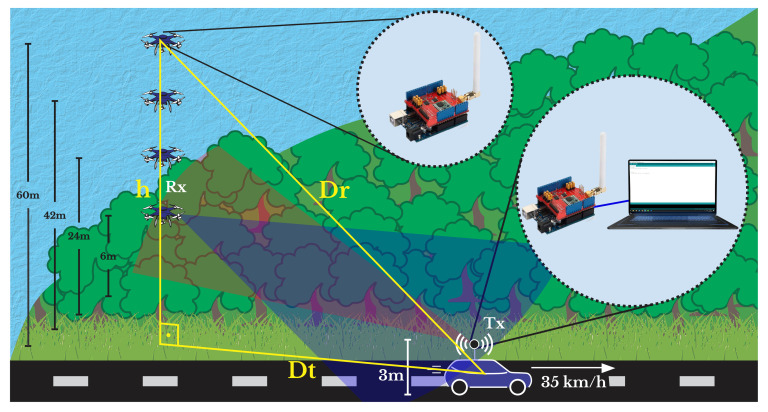
Measurement scenario.

At the Federal University of Pará (UFPA), measurements were conducted to obtain channel information in the 915 MHz frequency band using the LoRaWAN protocol. The measuring equipment consisted of two LoRa devices, a car, and an Inspire 1 model UAV. The chosen setup has a LoRa gateway attached to the UAV. In addition, the vehicle had a LoRa Dragino (an Arduino-based LoRa sensor) coupled to it at a 3-m height. Messages were sent and received, which enabled downlink and uplink communication analysis. The UAV position were between 6, 24, 42, and 60 m high. The vehicle was driven at a constant speed of 35 km/h. Figure 2 shows the measurement scenario.

**Figure 3:**
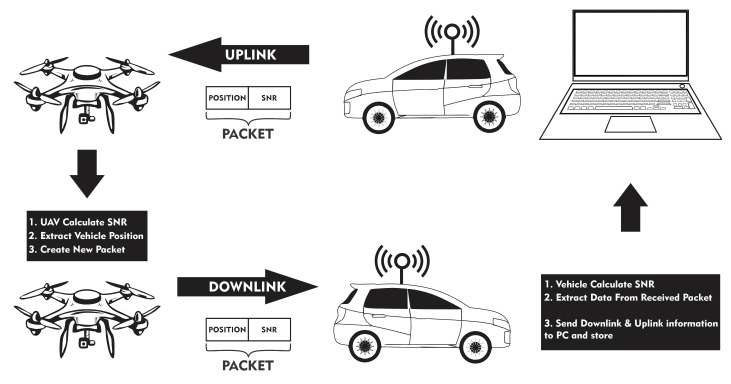
Transmission setup.

Where *h* is the UAV height from the ground, $D_{t}$ is the terrestrial distance, and $D_{r}$ is the radio distance between the car and the UAV. After defining the measurement scenario, it is necessary to define the transmission logic as shown in Figure 3.

First, the vehicle sends a packet with geolocalization value to the UAV gateway. The UAV Gateway calculates the uplink SNR and sends this new information to the vehicle. When the vehicle receives the data, it calculates the downlink SNR and then extracts packet information. Furthermore, a database of measurements is filled through serial communications between the endnode and the computer.

**Figure 4:**
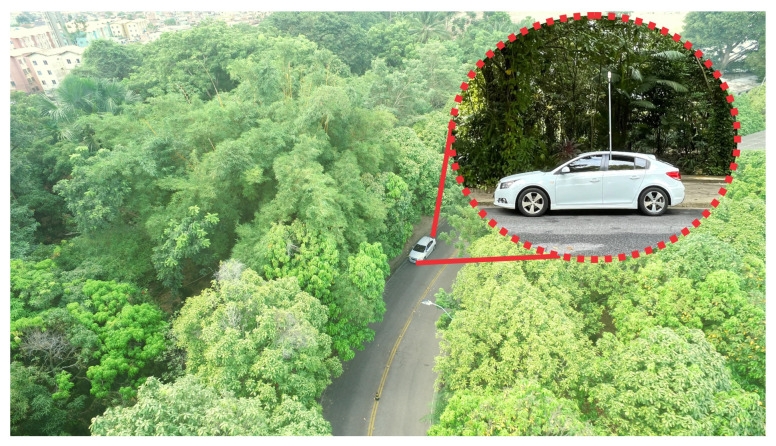
Vehicle in route.

**Figure 5:**
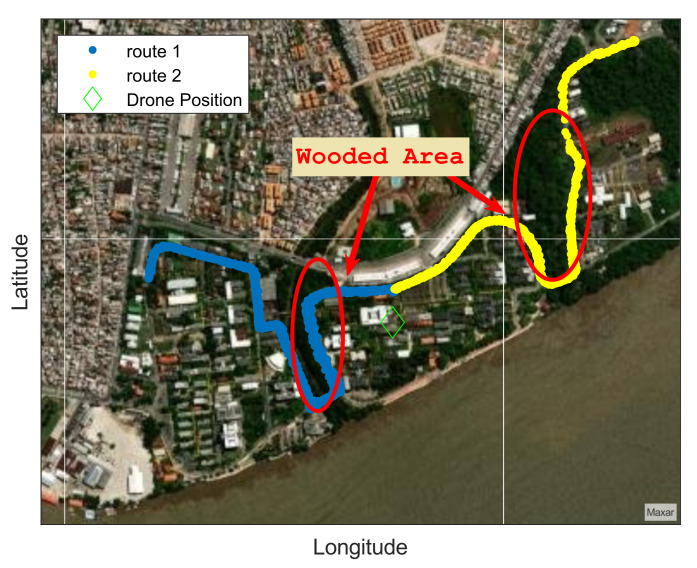
Measurement route.

The LoRa device was set up with the transmitter power equal to 20 dBm, 500 kHz bandwidth, 915 MHz operation frequency, and SF values switching between 8, 9, 10, and 11. The car, as shown in Figure 4, moves along two routes inside the UFPA to perform measurements, as visualized in Figure 5.

Route 1 is represented in blue, and is approximately 680 m long, while Route 2, in yellow, is approximately 1050 m long. The green marker represents the UAV position during the measurement campaign, and the red circles represent the wooded area with trees approximately 30 m high on each route. Each route was measured four times for each SF and drone height.

### 3.2. Data Preprocessing

After the measurement campaign, data were analyzed to calculate path loss and distance between the UAV and the vehicle, used as ANN input.

#### 3.2.1. Distance between UAV and Vehicle

The latitude and longitude values were used to calculate the distance between the car and the UAV through the Equation (1) based on the Haversine equation.

(1) $$\begin{array}{c} D_{r}=\sqrt{{\left(2r\arcsin\left(\sqrt{\sin^{2}\left(\frac{\varphi_{2}-\varphi_{1}}{2}\right)+\cos\left(\varphi_{1}\right)\cos\left(\varphi_{2}\right)\sin^{2}\left(\frac{\lambda_{2}-\lambda_{1}}{2}\right)}\right)\right)}^{2}+h^{2}}, \end{array}$$

where Dr is the radio distance between the UAV and vehicle in meters; *r* is the radius of the earth in meters (6,371,000 m); $\varphi_{1}$, $\lambda_{1}$ represent the coordinates (latitude, longitude) of point 1; $\varphi_{2}$, $\lambda_{2}$ represent the coordinates (latitude, longitude) of point 2; *h* is the UAV’s height from the ground.

#### 3.2.2. Path Loss Calculation

The path loss is another input of ANN and is calculated for each measured value through the Equation (2).

(2) $$Pl=10log(\frac{Pt}{Pr})-G$$

where Pl is the path loss at a given point in dB; Pt is the transmitted power in mW; *G* is the sum of transmission and reception gains in dBi, and Pr represents the received signal power in mW.

### 3.3. Collected Data

**Figure 6:**
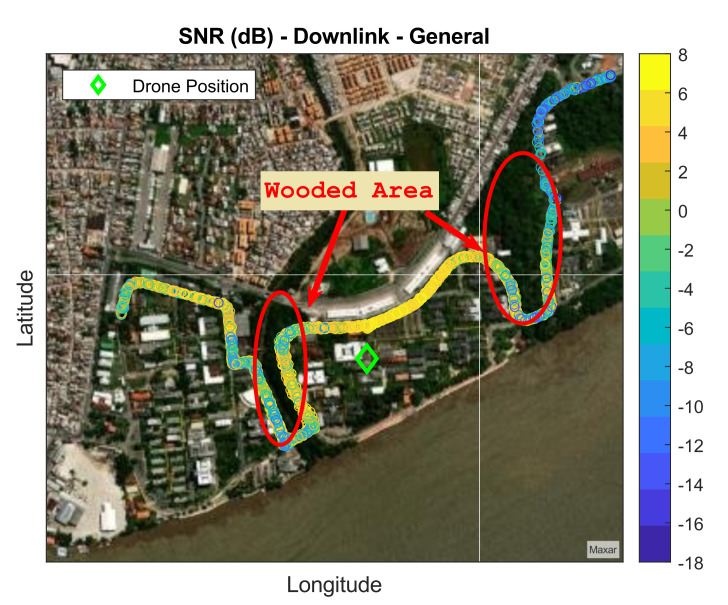
Downlink measured data.

After measuring each route four times for each SF and drone height, the total collected data were 3614 samples, divided between uplink and downlink. Figure 6 shows how the downlink SNR samples are distributed through the routes.

**Figure 7:**
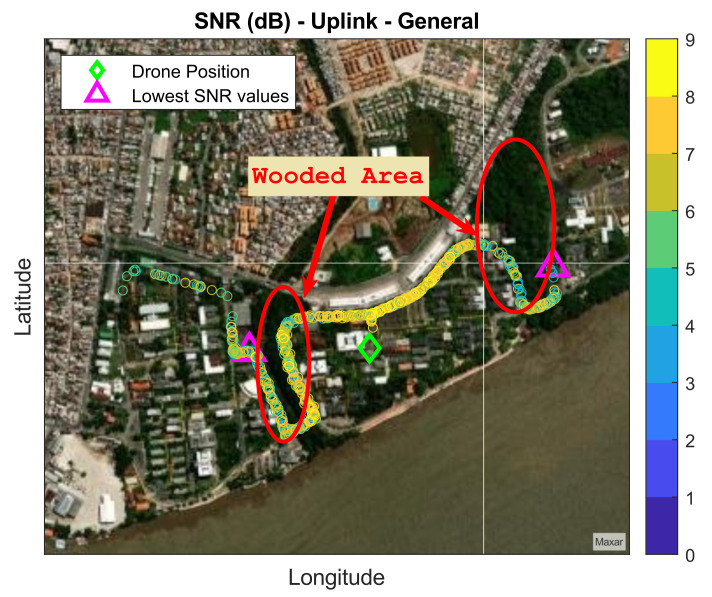
Uplink measured data.

The drone was located in the central region of the university, as shown in Figure 6. Observing Figure 6 the SNR decrease as the vehicle moves away. The SNR values ranged between −18 dB and 8 dB, with the smallest values represented in blue and the largest in yellow. The wooded area (red circles) shows a significant impact on transmission, mainly on Route 2, where the smallest SNR values were measured. Figure 7 shows measured results for SNR in the uplink case.

The SNR behavior remains the same as that in downlink communication: when the car moves away from the drone, the SNR reduces. However, in some stretches of both routes, the signal was drastically attenuated for the uplink, as evidenced by the significant reduction in its range. In Route 1, the trees perform a reduction in SNR until the signal becomes intermittent and weak; in Route 2, the wooded area demonstrates a more significant impact, since immediately after the vehicle goes behind, the signal disappears. The SNR values obtained in the measurement campaign remained between 0 dB and 9 dB for the uplink.

The lowest SNR values were found when the trees obstructed the transmission link between the transmitter (car) and receiver (drone), specifically when the car went near the vegetation-dense spots. For example, for a height of 42 m, the lowest values found were 0 dB in the positions illustrated by the magenta triangles in Figure 7. Only values of SNR equal to or greater than zero were obtained. Negative SNR values were not obtained because the SNR level was lower than the threshold defined in the datasheet [10], mainly because of the attenuation and spread phenomenon caused by trees.

**Figure 8:**
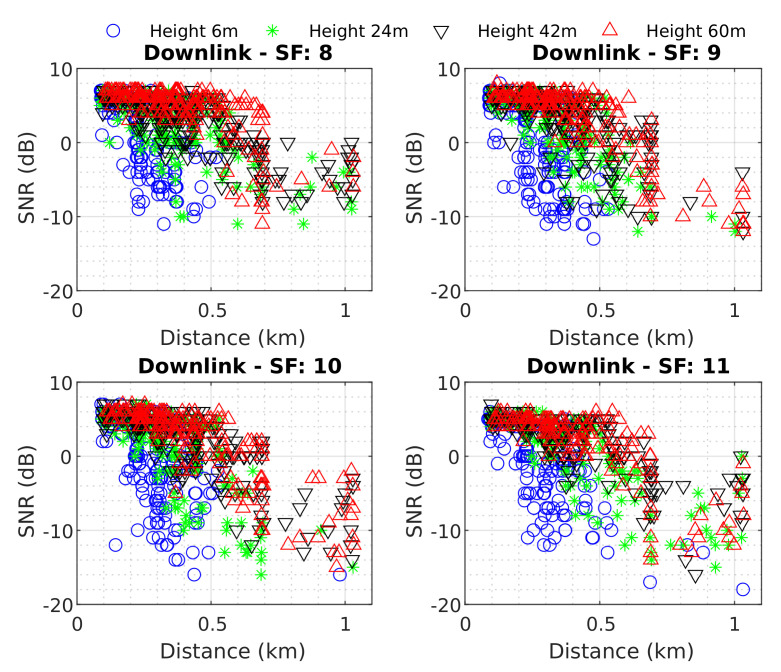
Downlink measured data SNR vs. Distance.

Figure 8 represents all collected data for the downlink scenario; we can observe the SNR decaying according to the drone’s height, its distance from the vehicle, and the SF used. Furthermore, through figure analysis, it is possible to observe that the coverage area increases according to these parameters, for example, drone height and SF. When the drone position was defined at a height of 6 m, the observed coverage/communication area reaches approximately 500 m $D_{r}$ (except for SF 11, which reached a greater distance). Additionally, for the other established height values, the gateway was capable of communicating with the endnode during all routes.

**Figure 9:**
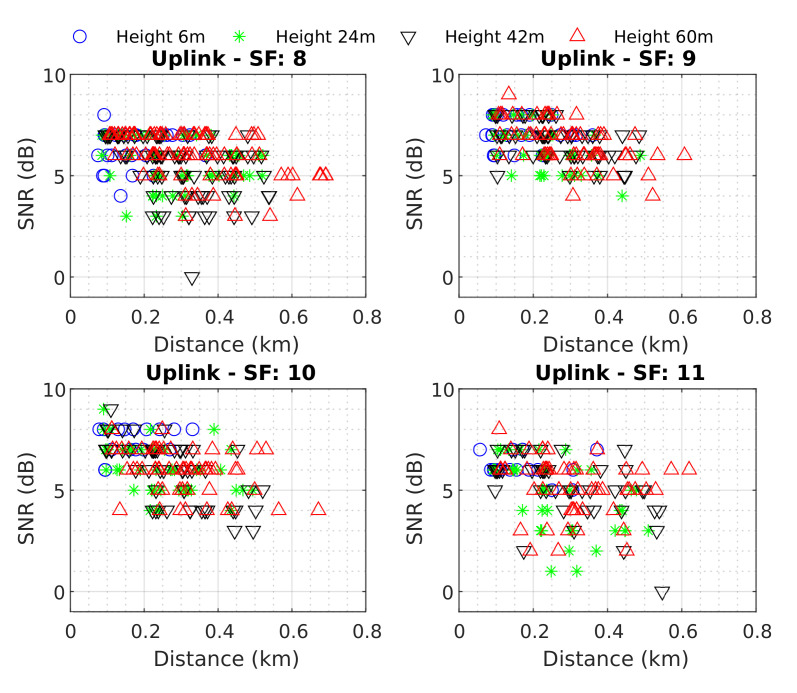
Uplink measured data SNR vs. Distance.

Figure 9 illustrates all the data obtained for the uplink scenario. When observing the drone positioned at a height of 6 m, it was possible to verify that it did not receive much data, obtaining a maximum $D_{r}$ range of 400 m. Considering the uplink as the main channel for most IoT applications, drone height is unfeasible. However, when analyzing other drone heights, the signal’s $D_{r}$ range was close to 600 m with similar behavior for all SFs, covering UFPA’s entire central region.

By analyzing Figure 8 and Figure 9, it is evident that downlink and uplink communication channels have different conducts. For example, in Figure 8 it is possible to observe a chaotic behavior, at low altitude in the range of 200 m to 500 m, then an accentuated decay in SNR values. In contrast, in Figure 9 the signal has a lower range and does not have well-defined behavior.

Therefore, from the results gathered in the experiments, the need to model propagation characteristics in both ways (gateway to endnode, and endnode to gateway) for mixed environments within the suburban context of Amazon rainforest cities became evident.

### 3.4. Artificial Neural Network

After data processing, according to the inputs: drone’s height, SF, radio distance, and propagation loss, a general regression neural network (GRNN) was trained to predict the SNR values for both uplink and downlink occasions.

#### 3.4.1. Generalized Regression Neural Network

The GRNN is a method for estimating the joint probability density function (PDF) given only a training set. The system is perfectly general because the PDF is derived from data without preconceptions regarding its shape. There are no problems if the function is composed of multiple non-Gaussian disjoint regions in any number of dimensions or simpler distributions [24]. For this ANN, the outputs were modelled as such:

(3) $$y_{j}=\frac{\sum_{i=1}^{n}\left(h_{i}\cdot w_{i}j\right)}{\sum_{i=1}^{n}h_{i}}$$

where, $y_{j}$ is the estimated function value; $w_{ij}$ is the desired output corresponding to the input training vector $x_{i}$ and output $y_{j}$; $h_{i}=e^{-\frac{D_{i}^{2}}{2\sigma^{2}}}$ is the output of a hidden layer neuron; $D_{j}^{2}={\left(x-u_{i}\right)}^{T}\left(x-u_{i}\right)$ is the squared distance between the input vector *x* and the training vector *u*; $u_{i}$ is the training vector, *i* is the center of neuron *i*; σ is a constant controlling the size of the receptive region.

The main advantage of GRNN over traditional training neural networks such as back-propagation neural networks (BPNN) is the shorter training time, which confirms its selection for modelling and controlling dynamic systems. In addition, GRNN has minor testing errors, which means that it has better generalization ability than BPNN [25].

**Figure 10:**
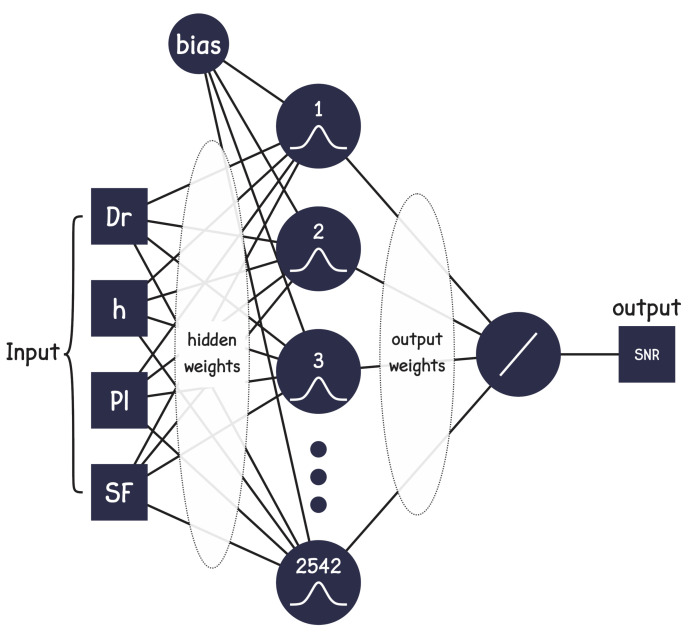
GRNN model.

The GRNN used in this study has the spread parameter, which determines how smoothe the function approximation is. The GRNN was set with spread value 8 to fit the data more smoothly (smaller values fit the data closely). The GRNN is composed of four inputs, two hidden layers, the first with 2542 neurons that use the radial basis transfer function. For the first layer, the number of neurons is equal to the number of training samples. The second layer has one neuron and uses the purelin transfer function, and the network has only one output, the SNR. Figure 10 illustrates the design of the GRNN network proposed in this study.

#### 3.4.2. Neural Network Training

The cross-validation process was used to train the GRNN to mitigate any bias caused by the particular sample chosen for training [26], with k = 10. In the traditional 10-fold cross-validation, data is split in 10 equal parts. The training process occurs in 10 iterations, for each iteration 9 folds (90% of measured data) are used in training and 1 fold (10% of measured data) is used in test [27].

**Figure 11:**
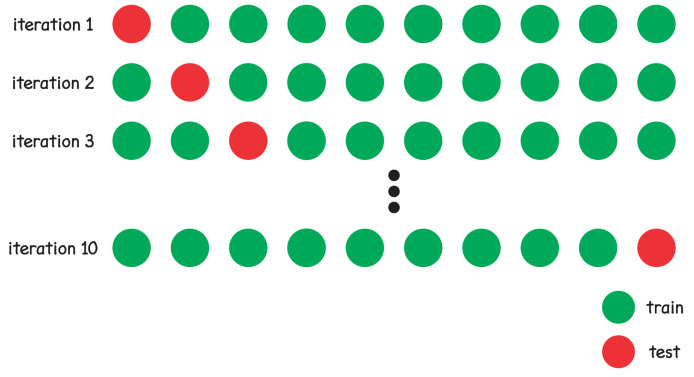
Cross-validation.

The process of 10-fold cross-validation training generates 10 different GRNN models. However the training process was repeated 100 times to ensure statistical variability, generating 1000 different GRNN models. The mean of error results are calculated to obtain GRNN model performance. Figure 11 illustrates the cross-validation process.

## 4. Results

This section presents and compares the baseline models and the proposed neural network. The best model was shown based on the obtained results.

Equations (4)–(6) represent the linear regression, sigmoidal, and power models employed as baseline models, respectively.

(4) y=a·x+b

(5) $$y=a\cdot x^{b}+c$$

(6) $$y=a+\frac{b-a}{1+a\cdot e^{-c\cdot x}}$$

### 4.1. Downlink

**Figure 12:**
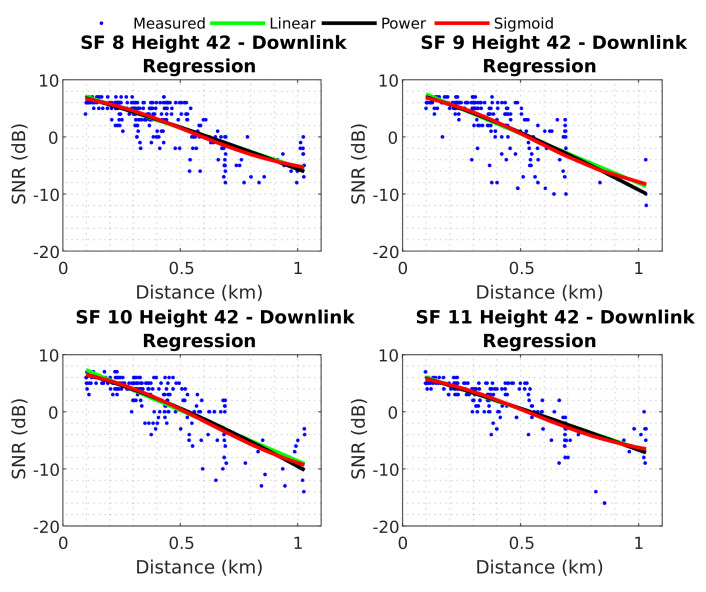
Baseline models downlink.

In this section, we describe the downlink scenario. There were three regressions over the measured data for each SF and height, totalling 48 regressions for the entire downlink scenario. Figure 12 displays the three regressions for each SF, in downlink, when the UAV is at 42 m high.

The curves in Figure 12 seem to fit the measured data well. Their root mean square error (RMSE) and error standard deviation (SD) values, together with their coefficients, are represented in the Table 1.

**Figure 13:**
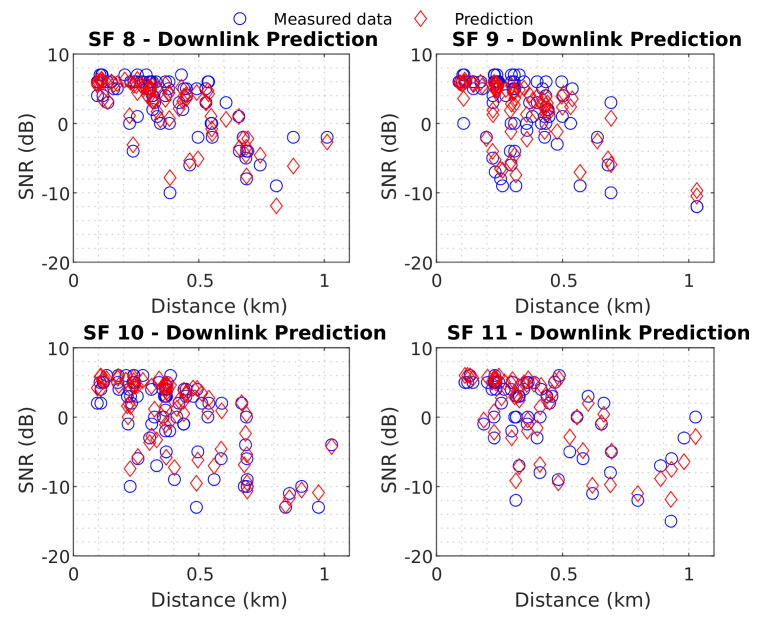
Downlink Best Neural Network.

The table demonstrates that most regressions presented similar results for each SF, with minor errors associated with SF 8 and a lower SD. The minimum RMSE value was 2.4249 dB for SF 8 (with linear regression), and the maximum was 3.3228 dB for SF 9 (with power regression). Appendix Table A1 presents a table with the regression coefficients and RMSE for all downlink measurements. After establishing a baseline, the effectiveness of the neural network was evaluated. The predictions of the best neural network are shown in Figure 13.

Figure 13 presents the results for the test data (which the ANN does not have access to during the learning process) in order to assess the predictive capability. It is possible to visualize the considerable accuracy presented by the model, as confirmed by the general 1.3817 dB RMSE and general 1.3784 dB error SD values. Table 2 summarizes the results of the best neural network model for each SF.

Table 2 shows that SF 9 obtained the greatest error, that is, 1.6623 dB, and SF 8 presented the minor, that is, 1.2322 dB. Finally, by comparing Table 1 and Table 2, it can be seen that the neural network model achieved better results than the established baselines for all SFs, with a difference of approximately 1 dB.

### 4.2. Uplink

**Figure 14:**
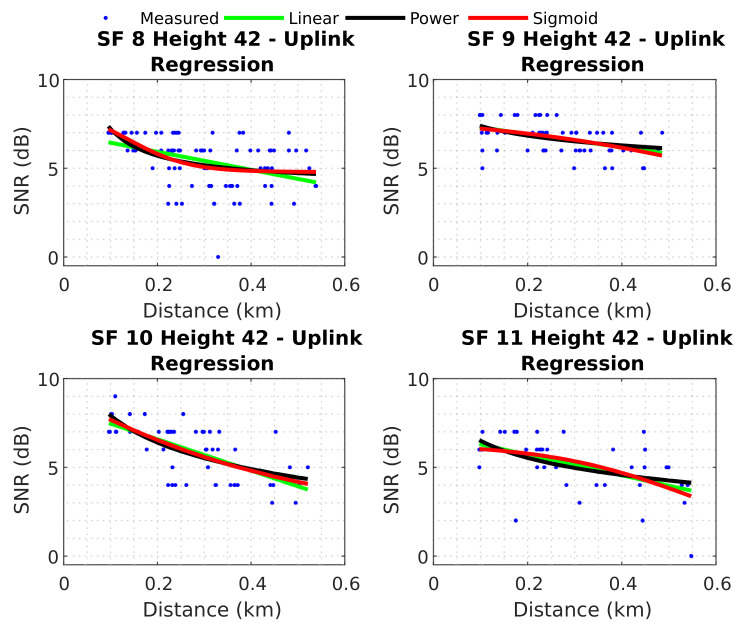
Baseline models uplink.

This section presents the neural network results obtained for the uplink scenario and establishes baseline regressions. Figure 14 shows the three regressions over measured data for uplink when the UAV is at 42 m high.

The fitted curves are shown in Figure 14 with RMSE below 2 dB for all three regressions and all SFs. Table 3 shows the RMSE and error SD for these uplink regressions with their respective coefficients. Appendix Table A2 presents a table with regression coefficients and RMSE for uplink measurements for each SF and the UAV’s height.

**Figure 15:**
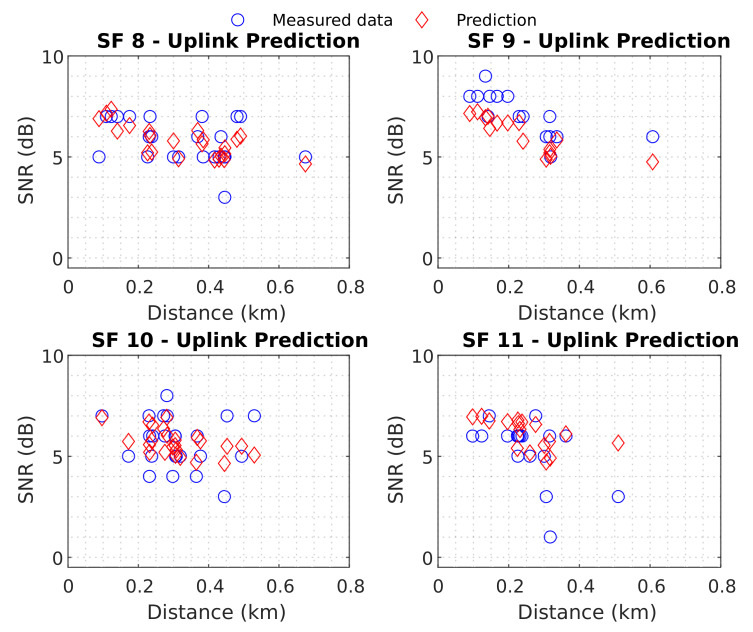
Uplink Best Neural Network.

In the uplink scenario, minor errors were associated with an SF of 9. The minimum RMSE value was 0.8245 dB for SF 9 (with linear regression), and the greatest RMSE value was 1.3233 dB for SF 11 (with power regression). Next, the results of the best neural network were compared with these baselines to verify their effectiveness. Figure 15 displays the predictions of the best neural network for uplink test data.

The neural network also presented a good predictive capability, with a general RMSE of 1.1102 dB and a general error SD of 1.1094 dB. For the best model, the RMSE remained below 1.4 dB for all SF. A slight difference between all SF was observed for the uplink. Table 4 contains the neural network results for each SF.

Table 4 shows that apart from the minor errors obtained for SF 8, the model was capable of predicting SNR satisfactorily in the uplink scenario with an RMSE of approximately 1 dB. Finally, comparing Table 3 with Table 4, it can be observed that the neural network performed worse than the regressions in SF 9 only. Nevertheless, the network presented promising results for both downlink and uplink communications. Therefore, the ANN use is viable for understanding how the signal will behave in a densely wooded suburban area, and the ANN-trained model for predicting transmission coverage and interference proposed in this paper should be able to optimize and aid future planning of wireless (LoRa-UAV) networks.

## 5. Conclusions

In this study, measurements were conducted at the Federal University of Pará to understand how the signal behaves in the communication between a UAV and moving vehicle located in a densely wooded suburban environment. The work showed that uplink and downlink channels exhibit different behaviors due to the difference in propagation media (vegetation and buildings, LOS, and NLOS) in the path of the electromagnetic wave between the transmitter and receiver. Another objective was to train a neural network model capable of predicting the measured SNR, helping to study the communication channel.

Data analysis showed an increase in range according to the established drone height and SF. Another highlight was the increase in reception sensitivity with an increase in the SF. The downlink communication distance reached the entire route, and this range was reduced to approximately 0.6 km for uplink.

Three baseline models and one neural network model were established. Their results showed that the neural network model surpassed all the regressions and presented the best results for both cases, obtaining a minimum RMSE of 1.6623 dB and a minimum error SD of 1.6730 dB in the downlink. A minimum RMSE of 1.3891 dB and error SD of 1.1706 dB were observed in the uplink analysis.

The significant downlink data variation showed the capability of neural networks to handle complex information because the prediction results were good for both downlink and uplink scenarios. The results obtained demonstrate the feasibility of using the model for channel analysis in this type of environment and its application to optimize the performance of LoRa-UAV networks.

In future work, applying the neural network model is expected to optimize the choice of SF and the UAV’s height to guarantee signal delivery with an acceptable SNR. Additionally, we would compare the GRNN models with other artificial intelligence techniques to verify which is best to solve the problem by comparing other metrics, such as computational cost and processing time.

## Figures and Tables

**Table 1:** RMSE values for downlink regression at 42 m high.

SF	Regression	Coefficients			RMSE	SD
	Linear	a = −13.7900	b = 8.4670		2.4249	2.4199
8	Power	a = −13.3800	b = 1.1470	c = 7.7340	2.4254	2.4154
	Sigmoid	a = 9.0050	b = −7.3260	c = 4.0420	2.3673	2.3575
	Linear	a = −17.3300	b = 9.2430		3.0311	3.0232
9	Power	a = −17.1800	b = 1.2590	c = 7.9320	3.0260	3.0102
	Sigmoid	a = 9.6200	b = −10.9600	c = 4.0290	3.0098	2.9941
	Linear	a = −17.6700	b = 9.0820		2.7153	2.7085
10	Power	a = −16.8200	b = 1.3350	c = 7.2610	2.6911	2.6775
	Sigmoid	a = 9.5280	b = −13.6100	c = 3.6310	2.6569	2.6435
	Linear	a = −13.8803	b = 7.4222		2.5616	2.5537
11	Power	a = −13.5700	b = 1.1070	c = 6.8800	2.5670	2.5511
	Sigmoid	a = 8.2700	b = −8.6359	c = 3.9258	2.5074	2.4918

**Table 2:** Downlink Neural Network Results.

SF	RMSE	SD
8	1.2322	1.2010
9	1.6623	1.6730
10	1.3511	1.2600
11	1.3183	1.2819

Source: The author.

**Table 3:** RMSE values for uplink regression at 42 m high.

SF	Regression	Coefficients			RMSE	SD
	Linear	a = −5.0930	b = 6.9470		1.3183	1.3104
8	Power	a = 0.4058	b = −0.9042	c = 3.9640	1.2729	1.2576
	Sigmoid	a = 8.4410	b = 4.7880	c = 15.4100	1.2576	1.2425
	Linear	a = −3.7390	b = 7.6750		0.8245	0.8171
9	Power	a = 79.8800	b = −0.0098	c = −74.3100	0.8496	0.8343
	Sigmoid	a = 0.0834	b = 8.0630	c = −3.3170	0.8296	0.8146
	Linear	a = −8.8290	b = 8.3430		1.1521	1.1411
10	Power	a = 67.4300	b = −0.0308	c = −64.4600	1.1660	1.1438
	Sigmoid	a = 2.1360	b = 23.6500	c = −2.9930	1.1550	1.1330
	Linear	a = −5.8280	b = 6.8770		1.2702	1.2560
11	Power	a = 124.9000	b = −0.0110	c = −121.6000	1.3233	1.2936
	Sigmoid	a = 0.0296	b = 6.3580	c = −6.2350	1.2753	1.2466

**Table 4:** Uplink Neural Network Results.

SF	RMSE	SD
8	0.8714	0.8905
9	1.1335	0.6295
10	0.9211	0.9409
11	1.3891	1.1706

## Data Availability

The authors reserve the right to not disclose private data set used in this study.

## Appendix A. Downlink Regressions

**Table A1:** Coefficient values for each downlink regression.

Regression	Height	SF	Coefficients (a,b,c)			RMSE	SF
Linear	6		−28.870	7.629		4.0472	4.0314
Power	6	8	34.670	−0.154	−43.290	3.9431	3.9121
Sigmoid	6		10.210	−3.687	14.440	3.8993	3.8688
Linear	6		−31.520	7.820		4.5076	4.4925
Power	6	9	−39.940	0.294	25.940	4.4773	4.4471
Sigmoid	6		9.462	−5.808	11.780	4.4680	4.4379
Linear	6		−26.920	6.290		4.7338	4.7178
Power	6	10	−54.420	0.179	41.500	4.6577	4.6261
Sigmoid	6		9.434	−5.929	12.130	4.6758	4.6441
Linear	6		−25.280	5.380		4.0767	4.0597
Power	6	11	−30.340	0.489	14.150	4.0206	3.987
Sigmoid	6		7.263	−16.150	5.301	4.1047	4.0703
Linear	24		−15.080	7.904		3.1730	3.1647
Power	24	8	−14.800	1.151	7.140	3.1779	3.1612
Sigmoid	24		8.608	−10.870	3.669	3.1681	3.1515
Linear	24		−22.900	10.000		2.9064	2.8983
Power	24	9	−23.000	1.230	8.505	2.8970	2.8809
Sigmoid	24		10.300	−14.980	4.450	2.8423	2.8265
Linear	24		−24.870	9.432		3.6520	3.6413
Power	24	10	−25.640	1.381	7.089	3.6237	3.6024
Sigmoid	24		10.050	−21.470	3.912	3.5858	3.5648
Linear	24		−18.790	7.989		3.1916	3.1809
Power	24	11	−19.190	0.925	8.603	3.2002	3.1788
Sigmoid	24		9.063	−11.030	4.778	3.0645	3.0440
Linear	42		−13.790	8.467		2.4249	2.4199
Power	42	8	−13.380	1.147	7.734	2.4254	2.4154
Sigmoid	42		9.005	−7.326	4.042	2.3673	2.3575
Linear	42		−17.330	9.243		3.0311	3.0232
Power	42	9	−17.180	1.259	7.932	3.0260	3.0102
Sigmoid	42		9.620	−10.960	4.029	3.0098	2.9941
Linear	42		−17.670	9.082		2.7153	2.7085
Power	42	10	−16.820	1.335	7.261	2.6911	2.6775
Sigmoid	42		9.528	−13.610	3.631	2.6569	2.6435
Linear	42		−13.880	7.422		2.5616	2.5537
Power	42	11	−13.570	1.107	6.880	2.5670	2.5511
Sigmoid	42		8.270	−8.635	3.925	2.5074	2.4918
Linear	60		−12.530	9.218		2.4909	2.4852
Power	60	8	−12.950	1.803	7.128	2.4199	2.4088
Sigmoid	60		9.971	−11.790	3.012	2.4206	2.4094
Linear	60		−17.890	10.530		2.2726	2.2671
Power	60	9	−17.510	1.841	7.320	2.0562	2.0462
Sigmoid	60		11.300	−22.900	2.844	2.0686	2.0586
Linear	60		−17.850	9.730		2.6396	2.6328
Power	60	10	−16.510	1.438	7.379	2.5955	2.5819
Sigmoid	60		9.972	−14.440	3.466	2.5609	2.5475
Linear	60		−16.400	8.690		2.8043	2.7953
Power	60	11	−15.370	1.665	5.990	2.7101	2.6928
Sigmoid	60		9.441	−15.950	3.148	2.6859	2.6687

## Appendix B. Uplink Regressions

**Table A2:** Coefficient values for each uplink regression.

Regression	Height	SF	Coefficients (a,b,c)			RMSE	SF
Linear	6		1.658	6.035		0.9550	0.9349
Power	6	8	4.347	2.025	6.168	0.9751	0.9335
Sigmoid	6		5.935	6.920	7.630	0.9752	0.9337
Linear	6		−1.275	7.382		0.7708	0.7539
Power	6	9	7.096	−0.027	−0.303	0.7903	0.7551
Sigmoid	6		7.682	4.769	2.859	0.7890	0.7538
Linear	6		4.063	6.666		0.7460	0.7222
Power	6	10	4.121	0.285	4.917	0.7701	0.7203
Sigmoid	6		0.322	8.087	7.291	0.7692	0.7194
Linear	6		−1.262	6.345		0.6295	0.6081
Power	6	11	−24.850	0.012	30.420	0.6438	0.5993
Sigmoid	6		6.593	5.889	16.930	0.6418	0.5974
Linear	24		−2.088	6.220		1.1710	1.1605
Power	24	8	−94.920	0.007	99.610	1.1602	1.1392
Sigmoid	24		17.180	−182.500	0.186	1.1826	1.1612
Linear	24		−6.536	7.860		0.9214	0.9106
Power	24	9	9.627	−0.122	−5.307	0.9172	0.8955
Sigmoid	24		8.249	5.273	12.330	0.9185	0.8968
Linear	24		−4.794	7.209		1.1188	1.1057
Power	24	10	0.014	−2.129	5.426	1.0495	1.0248
Sigmoid	24		9.350	5.546	23.570	1.0528	1.0281
Linear	24		−7.573	6.721		1.4725	1.4545
Power	24	11	−89.050	0.022	90.950	1.4522	1.4163
Sigmoid	24		−0.689	2.625	2.549	1.4456	1.4099
Linear	42		−5.093	6.947		1.3183	1.3104
Power	42	8	0.405	−0.904	3.964	1.2729	1.2576
Sigmoid	42		8.441	4.788	15.410	1.2576	1.2425
Linear	42		−3.739	7.675		0.8245	0.8171
Power	42	9	79.880	−0.009	−74.310	0.8496	0.8343
Sigmoid	42		0.083	8.063	−3.317	0.8296	0.8146
Linear	42		−8.829	8.343		1.1521	1.1411
Power	42	10	67.430	−0.030	−64.460	1.1660	1.1438
Sigmoid	42		2.136	23.650	−2.993	1.1550	1.1330
Linear	42		−5.828	6.877		1.2702	1.2560
Power	42	11	124.900	−0.011	−121.600	1.3233	1.2936
Sigmoid	42		0.029	6.358	−6.235	1.2753	1.2466
Linear	60		−3.559	7.190		0.9179	0.9130
Power	60	8	46.250	−0.023	−41.530	0.9247	0.9148
Sigmoid	60		3.316	21.100	−1.642	0.9215	0.9117
Linear	60		−5.322	8.180		0.8728	0.8664
Power	60	9	76.784	−0.018	−71.984	0.8816	0.8687
Sigmoid	60		4.069	27.424	−2.471	0.8741	0.8613
Linear	60		−3.635	7.058		1.0334	1.0244
Power	60	10	−30.258	0.038	34.800	1.0348	1.0168
Sigmoid	60		5.131	24.155	−5.361	1.0372	1.0191
Linear	60		−2.285	5.877		1.3721	1.3594
Power	60	11	−84.487	0.010	88.584	1.3622	1.3367
Sigmoid	60		8.323	4.908	21.020	1.3362	1.3112
